# Convolutional Neural Network Performance for Sella Turcica Segmentation and Classification Using CBCT Images

**DOI:** 10.3390/diagnostics12092244

**Published:** 2022-09-16

**Authors:** Şuayip Burak Duman, Ali Z. Syed, Duygu Celik Ozen, İbrahim Şevki Bayrakdar, Hassan S. Salehi, Ahmed Abdelkarim, Özer Celik, Gözde Eser, Oğuzhan Altun, Kaan Orhan

**Affiliations:** 1 Department of Oral and Maxillofacial Radiology, Faculty of Dentistry, Inonu University, 44210 Malatya, Turkey; 2 Department of Oral and Maxillofacial Medicine and Diagnostic Sciences, School of Dental Medicine, Case Western Reserve University, Cleveland, OH 44106, USA; 3 Department of Oral and Maxillofacial Radiology, Faculty of Dentistry, Eskişehir Osmangazi University, 26040 Eskişehir, Turkey; 4 Department of Center of Research and Application for Computer Aided Diagnosis and Treatment in Health, Eskişehir Osmangazi University, 26040 Eskişehir, Turkey; 5 Department of Electrical and Computer Engineering, California State University, Chico, CA 95929, USA; 6 Department of Oral and Maxillofacial Radiology, University of Texas Health Sciences Center at San Antonio, San Antonio, TX 79229, USA; 7 Department of Mathematics-Computer, Eskişehir Osmangazi University Faculty of Science, 26040 Eskişehir, Turkey; 8 Department of Oral and Maxillofacial Radiology, Faculty of Dentistry, Ankara University, 06100 Ankara, Turkey; 9 Ankara University Medical Design Application and Research Center (MEDITAM), Ankara University, 06100 Ankara, Turkey; 10 Department of Dental and Maxillofacial Radiodiagnostics, Medical University of Lublin, 20-001 Lublin, Poland

**Keywords:** sella turcica, artificial intelligence, CBCT, convolutional neural network

## Abstract

The present study aims to validate the diagnostic performance and evaluate the reliability of an artificial intelligence system based on the convolutional neural network method for the morphological classification of sella turcica in CBCT (cone-beam computed tomography) images. In this retrospective study, sella segmentation and classification models (CranioCatch, Eskisehir, Türkiye) were applied to sagittal slices of CBCT images, using PyTorch supported by U-Net and TensorFlow 1, and we implemented the GoogleNet Inception V3 algorithm. The AI models achieved successful results for sella turcica segmentation of CBCT images based on the deep learning models. The sensitivity, precision, and F-measure values were 1.0, 1.0, and 1.0, respectively, for segmentation of sella turcica in sagittal slices of CBCT images. The sensitivity, precision, accuracy, and F1-score were 1.0, 0.95, 0.98, and 0.84, respectively, for sella-turcica-flattened classification; 0.95, 0.83, 0.92, and 0.88, respectively, for sella-turcica-oval classification; 0.75, 0.94, 0.90, and 0.83, respectively, for sella-turcica-round classification. It is predicted that detecting anatomical landmarks with orthodontic importance, such as the sella point, with artificial intelligence algorithms will save time for orthodontists and facilitate diagnosis.

## 1. Introduction

The sella turcica (ST) is a saddle-shaped concavity located in the middle cranial fossa of the sphenoid bone and it houses the pituitary gland [1]. The ST anatomically consists of three parts: the tuberculum sellae, the pituitary fossa, and the dorsum sellae [2]. The sella turcica, teeth, and face parts originate from the neural crest cells. Therefore, anterior wall anomalies of the sella turcica are associated with anomalies in other structures, particularly in the frontonasal region [3]. Knowing the sella turcica morphology and distinguishing it from anomalies is essential as it contains the pituitary gland, and the sella point serves as a crucial anatomical reference in orthodontics [4].

In the literature, several studies have been conducted to reveal the typical morphology of the sella turcica. However, these studies were mainly carried out with two-dimensional radiographs such as lateral cephalometrics or cadaver examinations. In their retrospective study of 228 lateral cephalometric radiographs of healthy Nigerian individuals, Zagga et al. [5] examined the sella turcica in three subgroups: circular, oval, and flattened. They detected the oval shape at a rate of 83%, the circular shape at 11%, and the flattened shape at 6%. Ruiz et al. [6] examined the sella turcica in adult human skulls using CT and classified the shapes as “U”, “J”, and “shallow”, found in 48%, 41%, and 11% of the cases, respectively. Other classifications were based on the contours of the sella floor, the angles formed by the contours of anterior and posterior clinoid processes and tuberculum sellae, and the fusion of both clinoid processes known as sella turcica bridging [7]. Axelsson et al. [8] categorized the shapes of sella turcica into six main types: normal sella turcica, oblique anterior wall, double contoured sella, irregularity (notching) in the posterior part of the sella, pyramidal shape of the dorsum sellae, and sella turcica bridge, in which the normal morphology was found in 71% of males and 65% of females. In line with the study of Axelsson et al., Yassir et al. [9] reported a rate of 80.6% in girls and 71.4% in boys, whereas Shah et al. [10] and Alkofide [11] reported rates of 66.1% and 66.7%, respectively. 

In today’s world, artificial intelligence refers to any machine or technology that can simulate human cognitive skills such as problem-solving [12]. To understand artificial intelligence, it is essential to make a few definitions.

Artificial intelligence (AI) is defined as the ability of a machine to perform complex tasks that mimic humans’ cognitive functions, such as problem solving, recognition of objects and words, and decision making [13]. The objective here is to develop machines that can learn through data to solve problems.

Machine learning is the scientific study of algorithms and statistical models based on patterns and inferences to perform specific tasks [14]. The objective of machine learning is to make it easier for machines to learn through data so that they can solve problems without human interaction [12].

Deep learning is a subset of machine learning that uses layers organized as neural networks, similar to distributed communicating nodes, mimicking the synaptic structure of biological brains [14]. Deep learning aims to create a neural network that automatically identifies patterns to improve feature classification [12]. Convolutional neural networks (CNN) are a subset of deep learning and are well-suited to image classification [14]. In several disciplines, including engineering, agriculture, economics, medicine, and dentistry, there has been an increase in recent years in the amount of research based on AI algorithms [14,15,16,17,18].

Many specialists and general practitioners do not receive extensive training in radiographic image evaluation. Accordingly, they may not be competent at interpreting anatomical data, leading to dental practice difficulties, which necessitates a solution. The use of AI systems in radiographic interpretation provides offers advantages to the physician and may contribute to solving this problem. In addition, they can prevent misdiagnoses and incorrect treatment planning (due to workload, carelessness, or inexperience), as well as an unnecessary loss of time or workload in dentistry [19]. When the literature was searched, only one study using artificial intelligence-based models to segment the sella turcica was discovered [17]. In this study on lateral cephalometric radiographs, a U-Net architecture-based model and VGG19, ResNet34, InceptionV3, and ResNext50 transfer learning methods were compared, and it was determined that VGG19 and ResNet34 performed better than InceptionV3 and ResNext50. Similar to the previous work, in this study, the sella turcica was segmented and morphologically classified using a U-Net architecture and the GoogleNet Inception V2 transfer learning approach, and the sella turcica was classified using GoogleNet Inception V3. Additionally, superpositions in the lateral cephalometric images were eliminated in this study by using CBCT sagittal section images, thus improving the diagnostic performance of the model and adding a novel approach to the existing literature.

To the best of our knowledge, no prior studies used AI systems for the morphological classification of the sella turcica. To fill the research gap, the study presented here aimed to validate the diagnostic performance and evaluate the reliability of an artificial intelligence system based on the convolutional neural network method for the morphological classification of sella turcica in CBCT images. We anticipate that our proposed algorithm will make it easier for clinicians to obtain diagnostic information, which is our motivation in proposing this novel automated model for segmentation that uses a deep learning algorithm to automatically conduct sella turcica segmentation and classification.

## 2. Materials and Methods

### 2.1. Study Design

In this retrospective study, sella segmentation and classification models (CranioCatch, Eskisehir, Türkiye) were applied to sagittal slices of cone-beam computed tomography (CBCT) images, using PyTorch supported by U-Net and TensorFlow 1, and we implemented the GoogleNet Inception V3 algorithm. The study protocol was authorized by the Inonu University Non-Interventional Clinical Research Ethics Board (decision number: 2021/2753).

### 2.2. Data Sources

CBCT images obtained from individuals over the age of 18 (images of 188 patients taken for various reasons) were included in the study from the radiology archive of the Department of Dento-Maxillofacial Radiology of Inonu University School of Dentistry. Sex differences were not considered. CBCT images were saved as DICOM files. The open-source version 3.8 ITK-SNAP software (www.itksnap.org, accessed on 1 April 2022 ) was used to convert DICOM files to sagittal section frame images in JPEG format, and sections without sella turcica were extracted from the resulting images. Sagittal section images of this region were employed to evaluate the distance between the posterior clinoid processes in the axial mid-sagittal section. Despite the 0.2 mm interval between the sections, the images were selected at 1 mm intervals to avoid affecting the performance of the model because the images of neighboring sections would be similar to one another. In total, 1977 sagittal sections of CBCT images were used, comprising 199 sella-turcica-flattened images, 629 sella-turcica-oval images, and 1149 sella-turcica-round images, in the study. CBCT images were taken with the NewTom 5G CBCT device (Verona, Italy) with the following parameters: 110 kVp, 1–11 mA, and 3.6 s exposure time, with a 15 × 12 cm field of view (FOV) used for image acquisition.

### 2.3. Ground Truth

**Figure 1:**
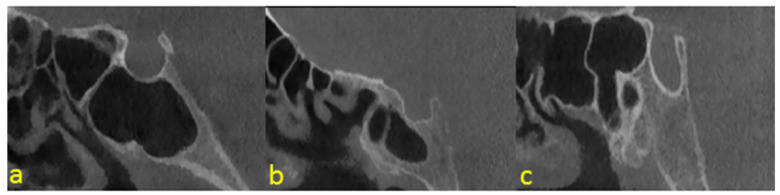
Examples of sella shapes: (**a**) round; (**b**) flattened; (**c**) oval.

Two dentomaxillofacial radiologists (Ş.B.D. and D.C.O.) with nine years and one year of experience, respectively, conducted ground truth labeling of sella turcica in the sagittal slices of CBCT images using the CranioCatch Labeling Tool (Eskisehir, Türkiye), with the joint decision for each label made using the polygonal box method. Images that could not be unanimously decided on were not included in the investigation. Sella turcica classification was carried out based on the sella shapes: flattened, round, or oval (Figure 1).

## 3. Models

### 3.1. Sella Turcica Segmentation Model

#### Pre-Processing Steps

A total of 1977 anonymized mixed-size sagittal slices of CBCT images were resized 512 × 512, to increase the visual quality. This study applied image enhancement techniques such as intensity normalization and contrast limited adaptive histogram equalization (CLAHE), and the images were separated into three categories: training, validation, and test groups. Images were randomly assigned to the training, validation, and test groups as follows:

*Training group:* 1587 (1587 labels)

*Validation group:* 195 (195 labels)

**Figure 2:**
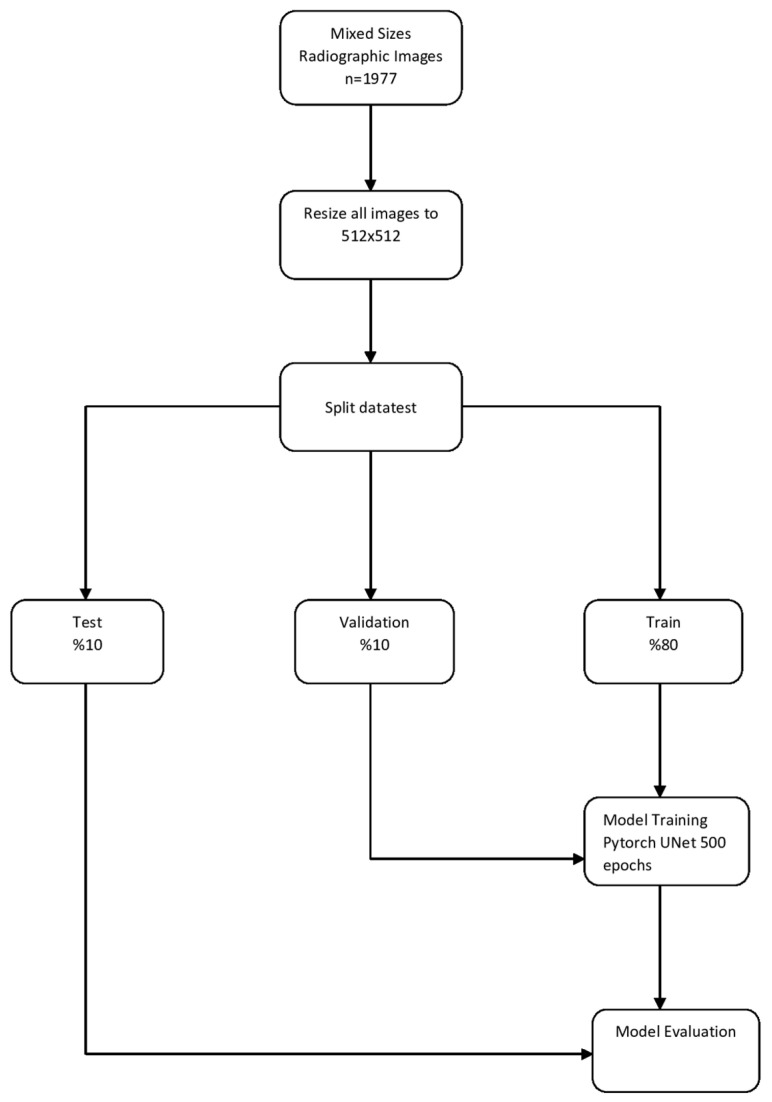
Model pipeline of automatic sella segmentation.

*Test group:* 195 (195 labels) (Figure 2)

### 3.2. Deep Convolutional Neural Network (CNN) Segmentation Model and Training

**Figure 3:**
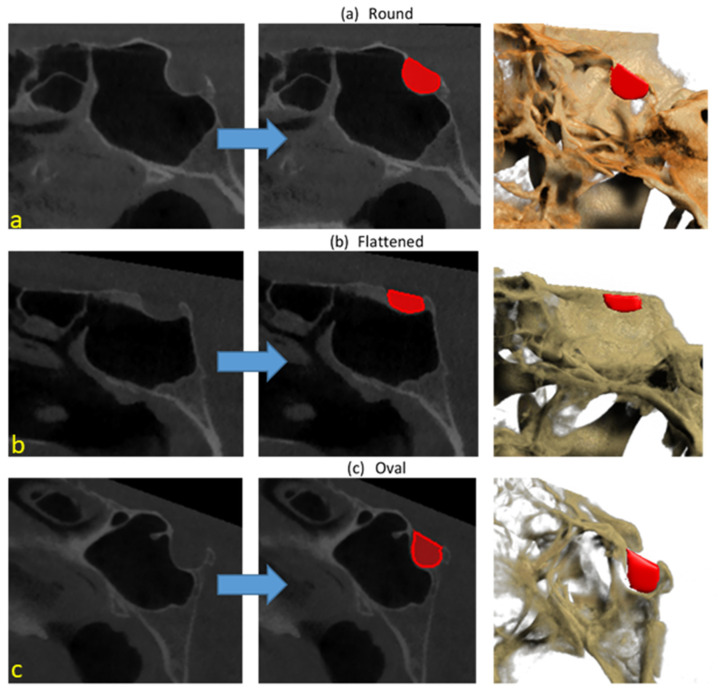
Automatic sella segmentation using deep-learning-based AI model: (**a**) round; (**b**) flattened; (**c**) oval.

Python, an open-source programming language (version 3.6.1; Python Software Foundation, Wilmington, DE, USA), was used to develop an AI algorithm. The PyTorch-library-supported U-Net algorithm was used to produce an AI model to segment sella turcica. The sella turcica segmentation model with U-Net supported by the PyTorch library was trained with 500 epochs and a 0.0001 learning rate. An AI model was produced (CranioCatch Labeling Tool, Eskisehir, Türkiye) to automatically segment the sella turcica model using CBCT sagittal images (Figure 2 and Figure 3).

### 3.3. Deep Convolutional Neural Network (CNN) Classification Model and Training

**Figure 4:**
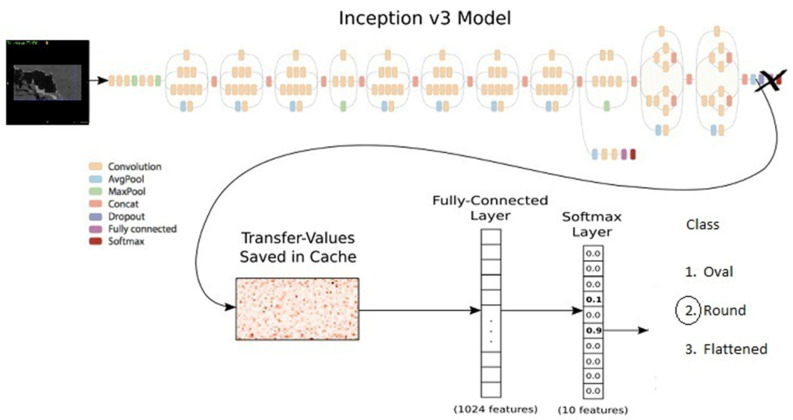
Deep CNN architecture of automatic sella classification.

The TensorFlow 1-supported GoogleNet Inception V3 algorithm was used to produce an AI model to classify sella turcica as flattened, oval, or round. The sella turcica classification model with TensorFlow 1, implemented using GoogleNet Inception V2 supported by the PyTorch library, was trained with 30,000 epochs and a 0.0001 learning rate. The Inception V3 model designed by Barker was followed [20] (Figure 4). The training process was carried out using the computer equipment of the Eskisehir University Faculty of Dentistry’s Dental AI Laboratory, including a Dell PowerEdge T640 Calculation Server (Dell Inc., Round Rock, TX, USA), Dell PowerEdge T640 GPU Calculation Server (Dell Inc., Round Rock, TX, USA), and Dell PowerEdge R540 Storage Server (Dell Inc., Round Rock, TX, USA). Details of the equipment’s features are provided in the Appendix A.

### 3.4. Sella Turcica Classification Model

A total of 1977 sagittal slices of CBCT images, comprising 199 sella-turcica-flattened images, 629 sella-turcica-oval images, and 1149 sella-turcica-round images, were resized to 512 × 512. Of these, 597 images were then split into training and test groups. Within the three classes, 179 images for training and 20 images for testing were used.

***Training group:*** 537 images, comprising 179 sella-turcica-flattened, 179 sella-turcica-oval, and 179 sella-turcica-round images.

***Test group:*** 60 images, comprising 20 sella-turcica-flattened, 20 sella-turcica-oval, and 20 sella-turcica-round images.

### 3.5. Performance Evaluation of AI Model

#### Metrics of Model Performance

A confusion matrix was employed to determine the model performance. The metrics used to assess the performance ST classification and segmentation model were as follows: 

**True Positive (TP):** The number of accurate segmentations or correctly classified models of the sella turcica class.

**True Negative (TN):** The number of correctly classified negative models of the sella turcica class. 

**False Positive (FP):** The number of sella turcica classes that were not segmented or correctly classified.

**False Negative (FN):** The number of wrongly segmented or classified sella turcica classes.

The performance metrics of the model were determined according to formulas using the number of TP, TN, FP, and FN cases, as below. 

**Sensitivity** (recall, true positive rate (TPR): TP/(TP + FN): Demonstrates how positive and negative values that are true are positive.

**Precision** (positive predictive value (PPV)): TP/(TP + FP): Indicates how accurately the positively predicted data were predicted.

**F1 Score:** 2TP/(2TP + FP + FN): A segmentation evaluation metric that effectively interprets the amount of overlap between the baseline truth and the prediction result.

**Accuracy:** (TP + TN)/(TP + FP + TN + FN): The degree to which a metric is accurate.

## 4. Results

The AI model based on deep learning achieved successful results for sella turcica segmentation of CBCT images. The developed AI model precisely segmented all test images. The sensitivity, precision, and F-measure values were 1.0, 1.0, and 1.0, respectively, for segmentation of sella turcica in sagittal slices of CBCT images (Table 1 and Figure 3).

The sensitivity, precision, accuracy, and F1-score were 1.0, 0.95, 0.98, and 0.84, respectively, for the sella-turcica-flattened classification. For the oval classification, they were 0.95, 0.83, 0.92, and 0.88, respectively. Lastly, the results were 0.75, 0.94, 0.90, and 0.83, respectively, for the sella-turcica-round classification. Table 2 and Table 3 present and summarize the AI model’s actual and predicted values. 

## 5. Discussion

In this study, an automated analysis was performed to classify the sella turcica morphology with three-dimensional CBCT based on a customized CNN deep learning algorithm, and the sensitivity was evaluated. The geometric center of the sella turcica is called the “sella point”. It is used as a cephalometric landmark to act as a reference point for evaluating the spatial position of both jaws relative to the cranial base [21]. In the present study, facilitation of the morphological classification of sella turcica by artificial intelligence aided the determination of the sella point, which is of excellent orthodontic importance. 

In the literature, several studies have been conducted to detect lateral cephalometric landmarks using artificial intelligence and machine learning algorithms [22,23,24,25]. Lindner et al. [26], in their study with the fully automatic landmark annotation (FALA) system, mentioned that the superposition of structures in two-dimensional radiographs such as lateral cephalometrics might increase the error rate in the determination of orthodontic reference points. The increasing use of cone-beam computed tomography (CBCT) in evaluating three-dimensional images and treatment planning has ruled out the limitations of conventional two-dimensional radiographs. Studies by Kochhar et al. [27] have shown that three-dimensional cephalograms created from CBCT images for the detection of cephalometric landmarks in patients with unilateral cleft lip and palate provide results that are more accurate and repeatable than digital lateral cephalograms. In orthognathic surgery, especially in individuals with skeletal malocclusion, rapid preoperative detection of anatomical landmarks in three-dimensional images obtained with CBCT, thanks to artificial intelligence, brings significant convenience to the physician. Neelapu et al. [28] detected 20 orthodontic reference points on 30 CBCT images in the MATLAB programming environment. They determined the overall detection rates as 64.16%, 85.89%, and 93.66% between the sensitivity ranges of 2, 3, and 4 mm, respectively. Although the Python programming language used in this study is similar to MATLAB with its features such as providing automatic memory management and having an interactive command system, it has the advantages of being a fully object-oriented language, supporting different coding styles, having a small command core that provides almost all the functionality a beginner will need, and being free software [29]. Lachinov et al. [30] on CBCT images; Lee et al. [23] used CNN-based regression systems for orthodontic landmark detection on lateral cephalometric radiographs. In this study, a CNN model based on the U-Net architecture was used, consisting of encoder, bottleneck, and decoder sections, which facilitated working with a limited number of datasets by using data augmentation techniques. For that reason, the use of such a model is preferred in applications such as biomedical image segmentation and classification. 

In their study aiming to detect orthodontic reference points in three-dimensional CBCT images using the MATLAB program, Montufar et al. [31] concluded that the irregular shapes and positional variations of the sella turcica complicate the detection of these points. Although in certain previous studies, the sella turcica morphology was classified from three-dimensional CBCT images [32], no studies had yet used artificial intelligence programs to classify these three-dimensional images. 

In this study, oval, circular, and flattened shape classifications were made during the introduction of the sella turcica morphology to artificial intelligence. During the classification process, it was determined that the anterior and posterior clinoid processes of some circular-shaped sella turcica were very close to each other and tended to merge. In the literature, the fusion of these clinoid processes is referred to as sella turcica bridging [33]. Many studies have been conducted on the relationship between sella turcica bridging and craniofacial anomalies, malocclusions, skeletal disorders, dental pathologies, and some syndromes [7,34,35,36,37,38,39,40]. While the importance of the sella turcica morphology has been revealed in many studies, we believe that this morphological classification study with artificial intelligence will make it easier for physicians to distinguish anomalies and pave the way for prospective patient studies to be conducted on sella turcica and other important orthodontic landmarks such as the porion, orbitale, and condylon.

This study has a few limitations. The data selected to introduce the morphological classification of sella turcica to the artificial intelligence program were obtained using a single CBCT device (NewTom 5G). Different machines and protocols may be studied to improve the accuracy of artificial intelligence-generated classification of the sella turcica morphology. Furthermore, a recent study recommended using a data pool consisting of multicenter data instead of data obtained from a single center for the generalization of results [41]. Another limitation was the relative decrease in image quality after the three-dimensional images of the sagittal section obtained with CBCT were converted to JPEG format using the semi-automatic ITK-SNAP application. As a final point to note, no power analysis was performed to determine the minimum sample size required during the pre-determination phase.

## 6. Conclusions

This CNN-based AI study showed a high percentage of accuracy. It is predicted that detecting anatomical landmarks with orthodontic importance, such as the sella point and shapes, using artificial intelligence algorithms will save time for orthodontists and facilitate diagnosis. Additionally, it is hoped that the present study will be a pioneer for prospective studies on the detection of anatomical landmarks in three-dimensional CBCT images using artificial intelligence programs.

## Figures and Tables

**Table 1:** Predictive performance measurement using the AI model for sella turcica segmentation in test data.

Model	TP	FP	FN	Sensitivity	Precision	F1 Score
Sella Turcica Segmentation	195	0	0	1	1	1

**Table 2:** Actual and prediction values of sella turcica classification model.

Classification of Sella Turcica		Prediction		
		Flattened	Oval	Round
Actual	Flattened	20	0	0
	Oval	0	19	1
	Round	1	4	15

**Table 3:** Predictive performance measurement using the AI model for sella turcica classification of test data.

	TP	FP	FN	TN	Sensitivity	Precision	Accuracy	F1 Score
Flattened	20	1	0	39	1	0.95	0.98	0.98
Oval	19	4	1	36	0.95	0.83	0.92	0.88
Round	15	1	5	39	0.75	0.94	0.90	0.83

## Data Availability

The data presented in this study are available on request from the corresponding author. The data are not publicly available because authors will be partially used in another study that is still being performed.

## Appendix A

The Eskisehir Osmangazi University Faculty of Dentistry’s Dental Artificial Intelligence (AI) Laboratory has advanced technology computer equipment including a Dell PowerEdge T640 Calculation Server (Intel Xeon Gold 5218 2.3G, 16C/32T, 10.4GT/s, 22M Cache, Turbo, HT (125W) DDR4-2666, 32GB RDIMM, 3200MT/s, Dual Rank, PERC H330+ RAID Controller, 480GB SSD SATA Read Intensive 6Gbps 512 2.5in Hot-plug AG Drive); PowerEdge T640 GPU Calculation Server (Intel Xeon Gold 5218 2.3G, 16C/32T, 10.4GT/s, 22M Cache, Turbo, HT (125W) DDR4-2666 2, 32GB RDIMM, 3200MT/s, Dual Rank, PERC H330+ RAID Controller, 480GB SSD SATA Read Intensive 6Gbps 512 2.5in Hot-plug AG Drive, NVIDIA Tesla V100 16G Passive GPU); PowerEdge R540 Storage Server (Intel Xeon Silver 4208 2.1G, 8C/16T, 9.6GT/s, 11M Cache, Turbo, HT (85W) DDR4-2400, 16GB RDIMM, 3200MT/s, Dual Rank, PERC H730P+ RAID Controller, 2Gb NV Cache, Adapter, Low Profile, 8TB 7.2K RPM SATA 6Gbps 512e 3.5in Hot-plug Hard Drive, 240GB SSD SATA Mixed Use 6Gbps 512e 2.5in Hot plug, 3.5in HYB CARR S4610 Drive); Precision 3640 Tower CTO BASE Workstation (Intel(R) Xeon(R) W-1250P (6 Core, 12M cache, base 4.1GHz, up to 4.8GHz) DDR4-2666, 64GB DDR4 (4 X16GB) 2666MHz UDIMM ECC Memory, 256GB SSD SATA, Nvidia Quadro P620, 2GB); Dell EMC Network Switch (N1148T-ON, L2, 48 ports RJ45 1GbE, 4 ports SFP+ 10GbE, Stacking).
